# Urinary excretion of low- and no-calorie sweeteners (LNCS) and associated food sources, as observed in the German cross-sectional KarMeN-study

**DOI:** 10.1007/s00394-025-03644-7

**Published:** 2025-03-24

**Authors:** Ralf Krüger, Bernhard Watzl, Benedikt Merz

**Affiliations:** Max Rubner-Institut, Department of Physiology and Biochemistry of Nutrition, Haid-und-Neu-Str. 9, D-76131 Karlsruhe, Germany

**Keywords:** LNCS, Urine, Sweetener, Cross-sectional, Mixed exposure

## Abstract

**Purpose:**

We aimed to quantify urinary excretion of LNCS (Low- and No-Calorie Sweeteners) and to identify LNCS-associated food consumption in Germany, with special emphasis on exposure to combinations of different LNCS.

**Methods:**

UPLC-MS/MS was used to quantify LNCS metabolites in 24-hour urine samples of 301 participants from the cross-sectional KarMeN (Karlsruhe Metabolomics and Nutrition) study. Dietary data were assessed via 24 h recall. Spearman rank correlation analysis and multiple linear regression models were used to investigate food groups that contribute to LNCS exposure.

**Results:**

Based on the number of samples with quantifiable urinary concentrations and the absolute excretion within a day, cyclamate (88% of samples), saccharin (44%), acesulfame (35%), and aspartame (32%) were most commonly consumed. The consumption of specific food groups, such as table sweeteners, light soft drinks, Radler, protein shakes, and stevia sweeteners, accounted for significant variations in urinary concentrations. Specific combinations of LNCS were observed for these food groups, as well as a considerable exploitation of LNCS-specific ADI (acceptable daily intake).

**Conclusion:**

Individuals who consume high amounts of specific LNCS-containing, processed foods are exposed to a notable mix of various LNCS. Since data on associations between mixed LNCS exposure and health are lacking, it is an urgent issue to evaluate the potential risks of consuming combinations of diverse LNCS rather than conducting risk assessments of single LNCS.

**Supplementary Information:**

The online version contains supplementary material available at 10.1007/s00394-025-03644-7.

## Introduction

Classical non-nutritive or low/no caloric sweeteners (LNCS) have been approved in the EU for several decades and are well established in the food industry. They are used especially in soft drinks, but also in processed convenience foods and in neat form as table sweeteners. With respect to ongoing discussions about strategies for reduction of sugar intake, LNCS become more and more relevant. Besides the important class of sugar alcohols (e.g. sorbitol, xylitol, erythritol), plus emerging low-caloric sugars such as allulose, classical LNCS still have a substantial market share, including sweeteners of the next generation [1, 2].

While intake of moderate LNCS quantities (below ADI) are generally considered safe by various food safety agencies, including EFSA, FDA, and German Federal Institute for Risk Assessment (*Bundesinstitut für Risikobewertung*, BfR), some concerns have emerged as to their potential long-term effects on health [3–6]. For instance, the use of cyclamate is prohibited in the USA and in South Korea since 1970, due to suspects of possible bladder carcinogenic effects [7]. Similar concerns have been raised regarding aspartame [8, 9]. Recently, the International Agency for Research on Cancer classified aspartame as possibly carcinogenic to humans (group 2B) based on limited evidence [10]. In one meta-analysis, LNCS intake was weakly associated with leukaemia, but not with overall cancer [11], whereas another systematic review revealed no evidence for association with any cancer type [12]. Other health-relevant associations were reported, for instance with cardiovascular diseases [13] or type-2 diabetes [14]. A recent umbrella review found evidence for higher risk of obesity, type 2 diabetes, all-cause mortality, hypertension, and cardiovascular disease incidence, but not for other diseases such as cancer or cardiovascular mortality [15]. In 2023, the WHO published an updated report on possible health effects related to the use of non-sugar sweeteners [4].

Energy intake from LNCS is negligible, suggesting their suitability to reduce sugar and total caloric intake [3, 16, 17]. However, body weight effects may differ between individual LNCS, and even body weight or adipose tissue gain have been observed [18–20]. For some sweeteners, an influence on taste perception [21, 22] and on food preferences in early life [23] was suspected, which would counteract their suitability as sugar substitute. Some reports suggest associations of LNCS intake during pregnancy with offspring obesity [24, 25], and with preterm risk in case of acesulfame K [26]. Most of those findings are controversial, and causal relationships have not been confirmed yet. Moreover, an influence on gut microbiota composition and subsequent metabolism has been increasingly and controversially discussed, especially at high doses which could lead to incomplete absorption in the small intestine, and subsequently to higher concentration in the colon [27–31]. Effects on down-stream glycaemic processes have been proposed as well [32, 33], but remain controversial.

In their 2023 released guideline, WHO advises not to use non-sugar sweeteners for weight control or for reducing the risk of noncommunicable diseases [5], a statement which has already provoked objections, related e.g. to inclusion of observational studies [34, 35]. Even more important, reliable conclusions about metabolic consequences of combined, regular LNCS intake are scarce, especially from controlled human studies. There is still a lack of information concerning synergistic effects of regular and in particular combined LNCS intake in considerable doses. Therefore, no conclusive risk assessment could be made so far for the exposure to a combination of different sweeteners.

Availability of reliable LNCS intake data is limited [1, 2], since it is generally difficult to cover them with classical dietary assessment tools used for nutrition surveys. Currently, exposure data or biomonitoring data for Germany are almost lacking, despite the obligation for a continuous surveillance, as required by EU law [36]. Data from other countries have limited informational value, especially from the North American region, where sugar-free sweetened beverages (light, zero) are much more common and have a significantly higher market share.

Some years ago, an LC-MS method for quantification of five LNCS in human urine was published [37], and urinary LNCS excretions were assessed in two cross-sectional studies from Greece and the Netherlands [38]. Data were reported also from China [39], and more recently from Taiwan [40] and once again from the Netherlands [41]. Comparable investigations for Germany are lacking so far. Therefore, we decided to use 24-hour urine samples from the cross-sectional KarMeN study to quantitate LNCS excretion and to identify LNCS-associated food consumption in south-west Germany, with special emphasis on the exposure to combinations of different LNCS.

## Methods

### Subjects and study design

The cross-sectional KarMeN (Karlsruhe Metabolomics and Nutrition) study was performed at the Max Rubner-Institut in Karlsruhe between 2011 and 2013. Study details are available elsewhere [42]. Briefly, healthy volunteers over 18 years old were eligible to participate. Exclusion criteria were smoking, acute or regular medication including hormonal contraceptives for women, illness requiring treatment, supplement use, and additionally for women pregnancy or breast-feeding. Recruitment procedures included direct communication with previous study participants, advertisements in local media (newspapers and radio), flyers, and word of mouth. Participation in the study was on a voluntary basis. 301 healthy male and female participants (18–80 years) visited the study center three times for a detailed characterization. Participants were examined by trained study personnel according to standard operating procedures, and anthropometric, clinical and functional parameters were assessed.

Participants collected their 24-hour urine, starting the morning prior to their 2nd study center visit. Collection bottles were kept in cool bags with cooling units throughout. Upon delivery of the 24-hour urine samples to the study center, the volume was recorded, 2 × 14 mL were centrifuged at 1850 g at 20 °C and aliquoted into small portions. Completeness of the 24-hour urine was checked by the para-aminobenzoic acid (PABA) recovery method using HPLC-UV [43, 44]. All samples were initially frozen at -20 °C for one day and then cryopreserved at -196 °C until analysis.

### LC-MS quantification of LNCS in urine

The method was developed based on a previously published method from Logue et al. as starting point [37], and adapted to UPLC-MS/MS conditions, i.e. lower flow rates and shorter run time. Moreover, additional analytes were added. A full list of analytes is provided in the compound table (Table S1).

An Acquity UPLC H-Class system coupled to a Xevo TQD triple quadrupole MS (Waters, Eschborn, Germany) was used. Compounds were separated on a RP-C18 column (Acquity BEH C18, Waters; 100 × 2.1 mm, 1.7 μm, with VanGuard pre-column) using a short gradient with a 7:3 mixture of methanol and acetonitrile as eluent and ammonium acetate buffer. MS detection was performed in negative ESI mode. Analyte-specific quantification was performed by MRM with time windows using up to two transitions per analyte. Isotope-labelled compounds were used as internal standards wherever possible, except for steviol and its glucuronide, for which glycocholic acid was used. Details of the MRM method are provided in Table S1. Further instrument parameters are provided in Table S2.

Samples were prepared by appropriate dilution (1:20, 1:200 in case of concentrations > 100 µM) and centrifugation (21.000 x g), without the need for laborious extraction procedures. Quantification was obtained by matrix-matched, internal calibration in the range 0 µM to 5 µM, corresponding to an upper limit of 100 µM in the original urine sample. Matrix-matched sample aliquots (1 µM and 3 µM) were used as quality control. Method validation was achieved as follows: Precision and accuracy (bias with respect to the calculated value) were tested using spiked matrix samples (2 levels). Within-day precision was determined batch-wise (*n* = 12), and controls of study sample sequences were used to determine day-to-day precision. Recovery and matrix effect were estimated in one experiment (2 levels, ISTDs 1 level; all *n* = 3). Matrix samples were spiked before or after sample preparation to calculate recoveries, and run together with matrix-free standards of identical concentration to calculate matrix effects. Lower limits of quantification (LOQ, LLOQ) and limits of detection (LOD) were estimated from dilution series using the following criteria: LOQ: Signal-to-noise ratio (S/N) > 10; LLOQ: S/*N* > 10, CV < 25%, bias < 25%; LOD: S/*N* > 3. The highest calibrator was defined as upper limit of quantification (ULOQ). Stability was extensively tested by Logue et al. [37] and therefore not assessed once again in the present work. Detailed information about chemicals, sample preparation and calibration can be found in Supplemental Information S3.

Steviol, other than steviol glucuronide, could not be detected in the urine samples. For hesperetin DC, strong interferences were observed upon analysis. Sucralose, advantame and neotame could not be validly quantified (S/*N* ≤ 10) in the vast majority of samples. Therefore, all five compounds were excluded from further analyses.

Results are given in mmol for 24 h total urinary excretion. Urine osmolality was determined by freezing point depression (Micro-Osmometer model 3MO, Advanced Instruments, Norwood, MA, USA). Glomerular filtration rate (GFR, ml/min) was measured based on 24 h urine creatinine clearance.

### Dietary assessment

Trained study personnel assessed the food intake of each individual (in g/day) for the day prior to their 2nd study center visit in a personal interview using 24-hour dietary recall with the software EPIC-Soft [45–47]. The 24-hour recall covers therefore the same period as the 24-hour urine collection. Participants used standard units (such as slice of bread, soup bowl), household measurements (such as tablespoon) and a picture booklet providing photographs of portion sizes for various foods to indicate the consumed amount per meal. Based on the EPIC-Soft food grouping, all reported foods were then summarized into 35 food groups for further analyses as already applied in other studies [48, 49] (Table S4). Based on the available information provided by the participants, the reported intake of table-top sweeteners was categorized to either ‘stevia sweeteners’ or ‘other sweeteners’. Furthermore, reported foods that were either reported as ‘light’, ‘zero’ or ‘reduced in sugar’ were allocated to additional food groups such as ‘light soft drinks’. As a consequence, the initial food group ‘beer’ was replaced by the more relevant food group ‘Radler’. The latter one commonly consists of a 50:50 mixture of beer with lemonade (incl. light versions) and is common in particular in German-speaking countries.

In order to identify further food groups that were not specifically covered by the initial 35 food groups, but may contribute to the intake of specific LNCS or LNCS in general, the reported individual foods of participants with urinary concentrations above 0.5 µmol/L per mOsm/kg urine for the sum of LNCS were reviewed in more detail. This led to the inclusion of the specific food group ‘protein shakes’ in further analyses.

### Estimation of LNCS intake via 24-hour urine excretion

To calculate the individual 24-hour urinary excretion of relevant LNCS, measured concentrations were multiplied by the corresponding recorded 24-hour urine volume for each participant. Intake of LNCS was then estimated based on available data on the respective LNCS excretion kinetics. Saccharin, acesulfame, and sucralose are not significantly metabolised in humans and, once absorbed, are excreted unchanged in urine and/or faeces [50].

Based on the assumption that 90% of consumed saccharin is excreted within 24 h with 80% excreted via urine and 20% via faeces [50, 51] we assumed 72% urinary excretion within 24 h. Thus, measured urinary excretion of saccharin was divided by 0.72 to calculate the respective dietary intake of saccharin. This principle was applied to all subsequent LNCS. Acesulfame is rapidly and almost completely absorbed. Maximum blood concentration was reached after 1–1.5 h and thereafter elimination occurred rapidly with a plasma half-life of 2–2.5 h. Only the parent compound could be identified in serum and urine, indicating that no significant degradation of acesulfame had occurred [2]. We assumed a 100% excretion within 24 h for acesulfame and for simplification that all acesulfame was ingested as acesulfame K (E950) and neglected the possible intake of the salt of aspartame-acesulfame (E962). Absorption of cyclamate from the gut is incomplete, and absorbed cyclamate is excreted via urine. For simplification reasons, we assumed a 22.5% urinary excretion of ingested cyclamate within 24 h [52]. For steviol glucuronide, we assumed that about 34% of the respective intake of its mother compound steviol glycoside is excreted as steviol glucoronide via urine within 24 h [53]. Bioavailability of intact neohesperidine DC is reported to be 21.8% [54]. For simplification reasons, we assumed that 100% of the absorbed amount is excreted within 24 h. Estimated excretion rates are summarized in Table S5. Aspartame is almost completely hydrolysed and in labelling experiments only 0.4 to 4% of radioactive metabolites were found in urine [55]. We performed exemplary calculations assuming conservative 0.4% urinary excretion of intact aspartame within 24 h to approximate the respective intake.

Evaluation of estimated intakes for each LNCS was done via relative coverage of established ADI values currently in use in the EU (Table S5). Here, individual LNCS exposure in mg/kg body weight was calculated by dividing the estimated individual intake by the respective body weight (in kg) for each participant. The resulting absolute LNCS exposure was then divided by the respective ADI values in the EU to derive the relative coverage.

### Statistical analyses

Since intact ranks are essential for some of the following analyses such as Spearman rank correlation analysis, metabolite concentrations with a S/*N* ≤ 10 were manually set to zero, since these measures showed a high analytical uncertainty, including their ranks.

In a preliminary selection step, we used Spearman rank correlation analyses to identify those food groups that are significantly positively correlated with each of the measured urinary LNCS metabolite concentrations or the sum of them, respectively. For those food groups that were significantly correlated with urinary metabolite concentrations, we investigated in a second step associations between metabolite concentrations as dependent and the identified food groups as independent variables in multiple linear regression models, again for each metabolite and the sum of metabolites. Since food groups and urinary metabolite concentrations were not normally distributed these variables were univariate Box-Cox transformed to approach normality [56] and z-standardized to a mean of 0 and a standard deviation of 1 to ensure comparability between food groups or metabolite concentrations, respectively, prior to regression analyses. For the Box-Cox transformation, we used an optimization step to find the best transformation parameter (exponent lambda) between 0 and 2 for appropriate transformation of each variable.

Explained variances for each food group were calculated as the difference of adjusted R² when taking the food group of interest out of the multiple model [48, 57–59]. Each parameter was taken out of the model and the difference to the complete model was calculated. The sum of single contributions was lower than R^2^ of the complete model, pointing to interaction effects. To account for this, single contributions were normalized to the sum of all explaining factors. We investigated potential confounders, including age, sex, fat mass index as a measure of body composition, glomerular filtration rate, and total energy intake, by incorporating them into our model. None of these variables were statistically significant and were therefore removed from the model.

All statistical analyses were performed using SAS Version 9.4 (SAS Institute, Cary, NC, USA) with p-values < 0.05 considered statistically significant.

## Results

**Fig. 1:**
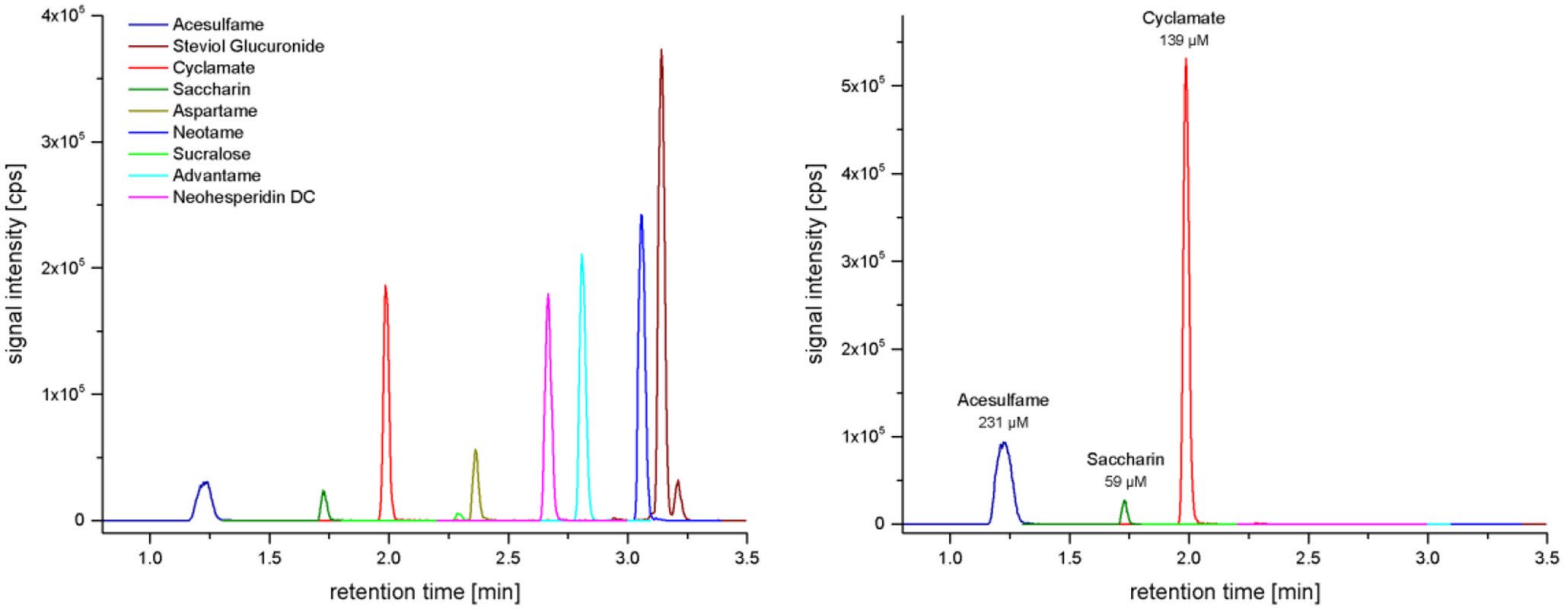
Example Chromatograms: (left panel) Standard mixture 50 µM (matrix spike); (right panel) Study sample from KarMeN showing substantial, combined excretion of three LNCS

A matrix-matched UPLC-MS method with simple sample preparation and a very short 3-minute gradient (turnover 7 min) was established and evaluated thoroughly. The final method proved to be robust and reliable. Precision was in the range of 3–16%, recoveries were between 76 and 97% and LOQ were in the nM range. Validation data are given in the Table S6. Example chromatograms of a spiked standard sample and a KarMeN study sample are shown in Fig. 1.

The study population consists of 301 participants, 172 men (57.1%) and 129 women (42.9%) with an age range from 18.9 to 80.4 years. General characteristics of the study participants stratified by sex are shown in Table 1.

**Table 1:** General characteristics of the study population

	Men	Women
N	172	129
Age (years)	44.4 ± 17.9	51.7 ± 15.0
BMI (kg/m²)	24.4 ± 2.7	23.2 ± 2.9
Body fat (%)	23.9 ± 6.6	34.8 ± 6.8
Fasting glucose (mg/dl)	86.6 ± 8.2	84.9 ± 7.5
GFR (ml/min)	108.5 ± 17.5	116.2 ± 19.8

Values are given as absolute numbers or arithmetic mean ± standard deviation

Of the analysed LNCS, quantitative urinary concentrations (S/*N* > 10) could be obtained for cyclamate (in 88% of the analysed samples), saccharin (44%), acesulfame (35%), aspartame (32%), neohesperidin DC (17%) and steviol glucuronide (16%). In contrast, sucralose (> 99%), neotame (97%) and advantame (91%) could not be quantified in the vast majority of the analysed samples. Accordingly, mean urinary concentrations were highest for cyclamate, saccharin, and acesulfame. Total 24 h urinary LNCS excretion of our study population is given in Table 2 and Table S7. We did not observe significant differences between men and women (data not shown).

**Table 2:** 24h urinary LNCS excretion (in mmol, mean ± SD) in total and stratified by quartiles of total urinary LNCS excretion

Variable	*N* = 301	Q1 (*n* = 75)	Q2 (*n* = 75)	Q3 (*n* = 76)	Q4 (*n* = 75)
Σ LNCS	0.06±0.21	0.60±0.36	2.47±1.06	12.0±6.14	294±596
Acesulfame	0.03±0.12	0.05±0.14	0.26±0.73	5.22±6.93	133±271
Steviol glucuronide	0.00±0.00	0.04±0.13	0.14±0.34	1.48±3.40	0.91±3.53
Cyclamate	0.02±0.09	0.12±0.13	0.39±0.68	1.66±3.81	114±288
Saccharin	0.01±0.04	0.11±0.25	1.21±1.13	3.24±4.70	45.9±122
Aspartame	0.00±0.00	0.15±0.21	0.17±0.27	0.16±0.19	0.14±0.30
Neohesperidin DC	0.00±0.00	0.01±0.03	0.01±0.03	0.01±0.02	0.02±0.09

### Explained variance by contributing food group

**Fig. 2:**
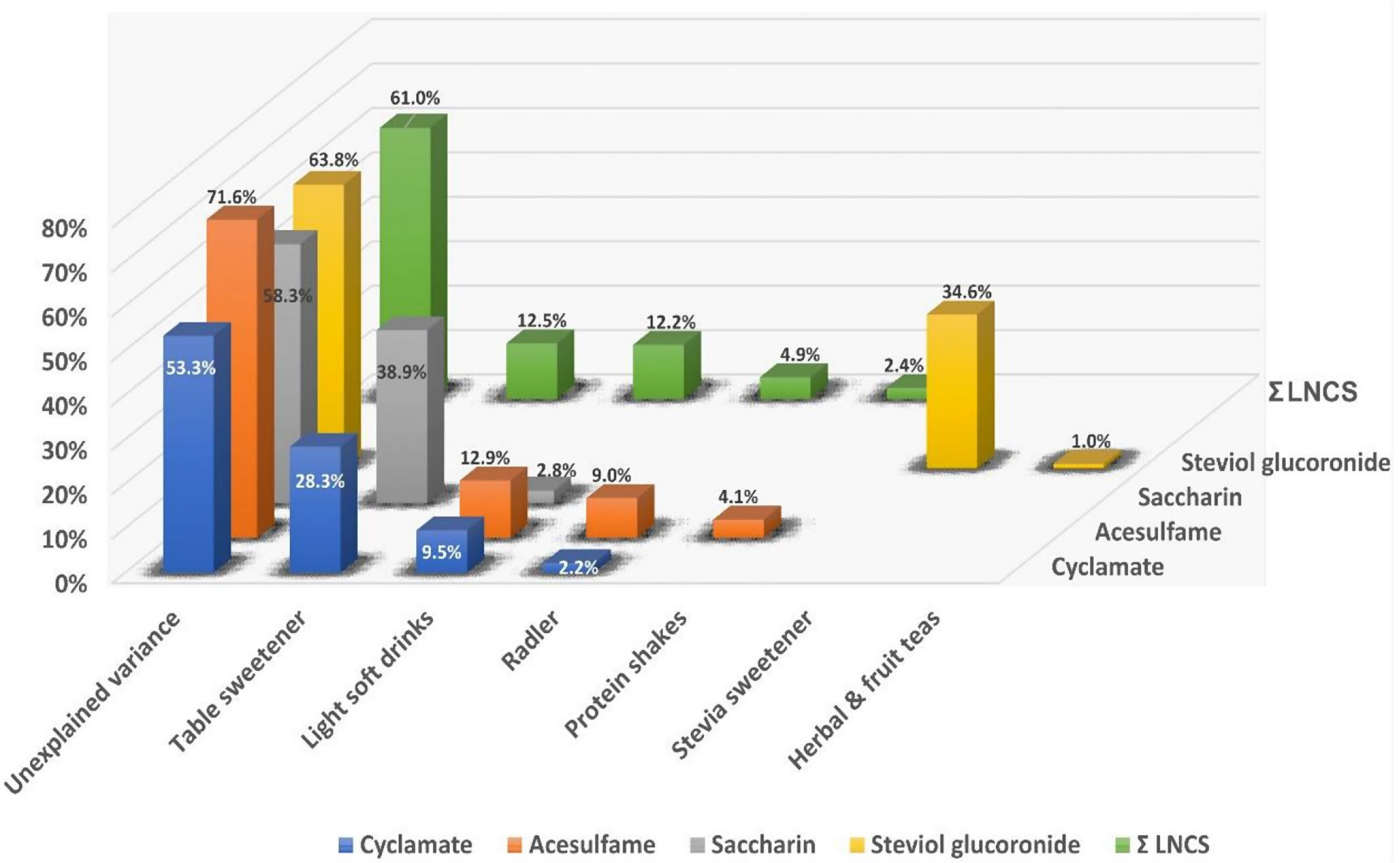
Explained variances for the single urinary LNCS concentrations and their sum by contributing food groups (only food groups contributing at least 1% to the explained variance are shown)

For cyclamate, food groups `table sweeteners´ (18.1%), `light soft drinks` (7%) and `Radler´ (2.3%) explained a total of 27.4% of the observed variation, whereas for saccharin, mainly `table sweeteners´ (38.9%) and to a lesser extent `light soft drinks´ (2.8%) were associated, explaining up to 41.7% observed variation. Acesulfame was associated with `light soft drinks´ (12.9%), `Radler´ (9.0%), and `protein shakes´ (4.1%) which altogether explained 28.4% of the observed variation. Steviol glucuronide as the main urinary metabolite of steviol glycoside was associated with food groups `stevia sweetener´ (34.6%) and `herbal & fruit teas´ (1.0%) and an overall explained variance of 36.2% in the model. With regard to the sum of all LNCS metabolites, the multiple regression model explained 39.0% of the observed variance, with `table sweeteners´ (12.5%) and `light soft drinks´ including `Radler´ (17.1%) explaining the largest part (Fig. 2). We also observed a weak but statistically significant correlation between acesulfame and ‘curd and fresh cheese’. Compared to the explained variance of other food groups, contribution of ‘curd and fresh cheese’ to the overall variance (0.6%) was neglected.

### Exposure to combinations of LNCS

**Fig. 3:**
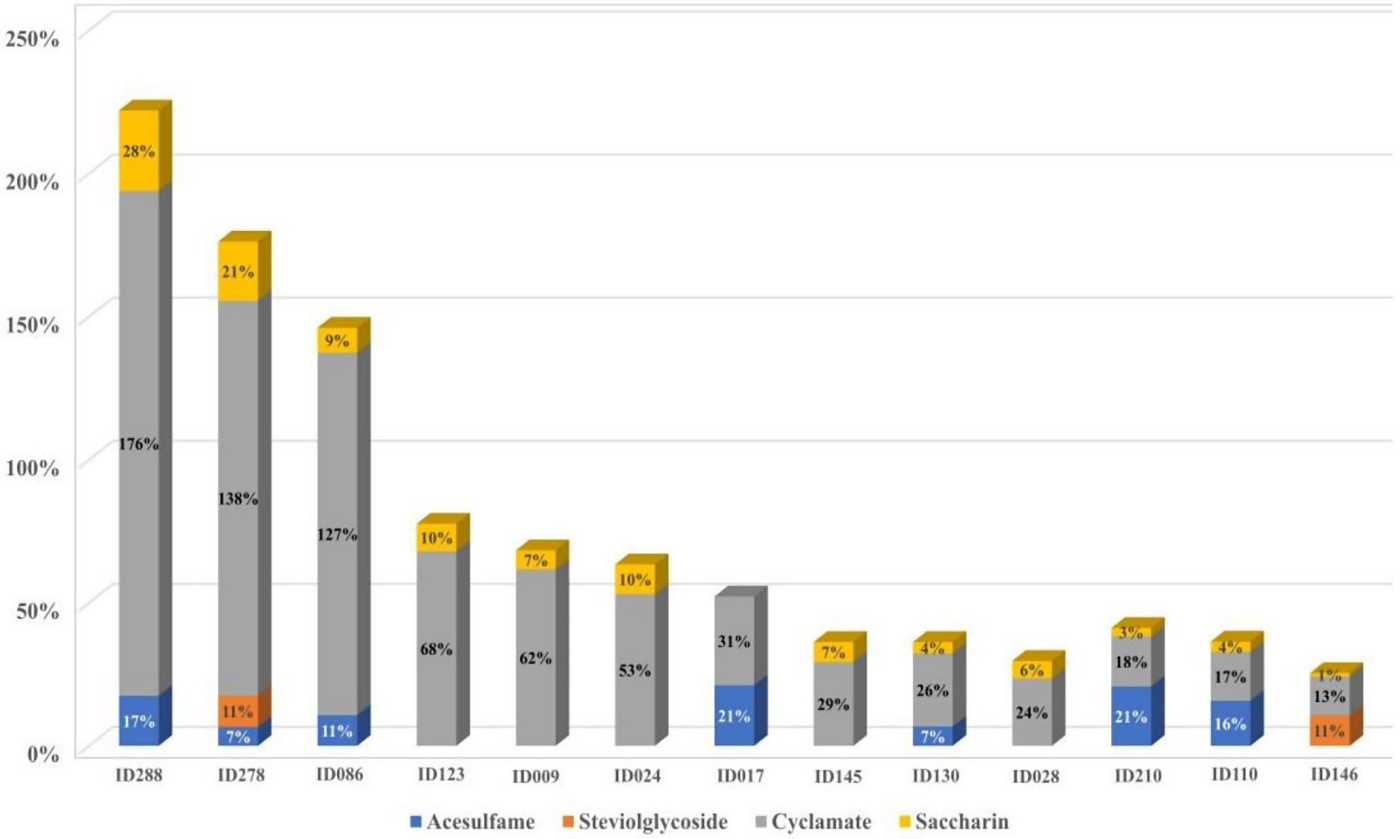
Percentage coverage of respective ADI values on an aggregated scale for participants with a significant mixed LNCS exposure

In our study population, we observed the exposure to combinations of different LNCS. Based on the estimation of LNCS intake, and taking into account the current EFSA ADI values, we calculated that cyclamate had the highest exposure among LNCS, with three participants exceeding the ADI (176%, 138%, 127%, see Fig. 3). Significant exposure was also observed for acesulfame (max. 21.2% respective ADI exploitation), steviol glycoside (max. 21.9%), and saccharin (max. 28.1%). For aspartame (scenario 0.4%), we estimated a maximum ADI exploitation of 4.6%. The number of participants with respect to ADI exploitation is given in Table S8. We observed that high consumers had intake of doses higher than 10% of the respective ADI for more than one LNCS. This resulted in a notable mixed exposure for these participants. We identified a comparably high exposure to combinations of various LNCS for a few participants. E.g., we observed an exceedance of > 10% ADI for at least two LNCS in six participants, and for two of those participants, an exceedance of more than 10% of the ADI for three LNCS. Furthermore, we observed an exploitation of more than 20% ADI for two LNCS in two participants.

## Discussion

### Method performance

The optimized LC-MS method was successfully validated and proved to be robust. In comparison to the initial method [37], run time was halved to about 7 min at reduced column size and flow rate, which saves analysis time and solvent consumption. Modifications of separation parameters (slightly acidic gradient, mixed organic solvents) supported chromatography and robustness. Moreover, the LNCS panel was extended by adding not only neohesperidin DC, but also peptidic LNCS. Despite the fact that these compounds are almost completely metabolized to corresponding amino acids in humans, we nevertheless detected traces of the intact precursor, especially aspartame. Obviously, a few percent of peptidic LNCS evade from being digested, and are excreted without metabolic conversions. To our knowledge, this is the first study to report excretion of intact aspartame. In the light of recent safety discussions [4, 10], coverage of aspartame excretion and subsequent estimation of intake is highly relevant. Further investigations are underway to substantiate this finding. We conclude that trace analysis of aspartame, neotame and advantame should be possible using a LC-MS instrumentation with ultrahigh sensitivity. Very recently, an alternative, albeit longer HILIC-based method was described, including urinary LNCS and sugars, but not peptidic LNCS [60].

### LNCS excretion as compared to other biomarker studies

Since the assessment of the dietary intake covers exactly the collection period of the 24-hour urine samples, ingested LNCS amounts can be inferred and results can be compared with results from other countries. In an adult cohort from the Netherlands, a similar LNCS consumption pattern was found in 24 h-urine samples [38]. Most prominent LNCS were saccharin, acesulfame and cyclamate, in accordance with our findings. However, detected quantities were higher in the Netherlands, and relative contributions of LNCS were different. The order was as follows (median (IQR) [mg/day]): cyclamate 0.13 (0.05–1.16) > acesulfame 0.00 (0.00-1.02) > saccharin 0.00 (0.00-0.70) in our study; acesulfame 5 (0–21) > cyclamate 1 (0–11) > saccharin 0 (0–4) in the Dutch study. The different abundance of acesulfame and cyclamate might be related to regional differences, even though these two study areas are only a few hundred kilometres apart. In addition, market shares change over time, but both studies have been conducted in a similar time period before 2011. It is also likely that study characteristics such as age, socioeconomic status or national food preference might contribute.

Considering the percentage of LNCS consumers, instead of LNCS mean or median quantities excreted, the order is slightly different. In our study, cyclamate was still the most abundant LNCS (88% of participants; acesulfame and cyclamate < 50%), whereas Logue et al. [38] reported 90% for saccharin, 74% for acesulfame and 68% for cyclamate. This order agrees with data from Greece, presented in the same article, where saccharin was by far the most abundant LNCS: saccharin (88%) > acesulfame (51%) > cyclamate (34%). Obviously, saccharin consumption seems to be lower in our south-west German study population, compared to other European regions.

A second, recent study in the Netherlands from Buso et al. [41] reported comparable mean 24 h-urinary excretions. They found saccharin (in 97.9% of the samples), acesulfame (83%), steviol glucuronide (67%) as the most frequently detected LNCS in their study, in accordance with the earlier study from the Netherlands discussed before [38], but in contrast to our study. Within the KarMeN study, cyclamate was the most prominent LNCS being present in 88% of the samples, whereas steviol glucuronide was quantifiable in only 16% of the samples. This could be partly explained due to different criteria for quantification limits applied in both studies and differences regarding the LNCS used in the food industry in Germany and the Netherlands.

In a study from Tianjin, China, three main LNCS saccharin, cyclamate and acesulfame were found in 100% of the investigated urine samples [39]. In another recent publication, LNCS urinary excretion data from Taiwan were presented [40]. Unfortunately, excretion rates are given as ng/mg creatinine in both studies, preventing direct comparison with our results. Moreover, two of the most abundant LNCS (saccharin and cyclamate) were not covered in the Taiwanese study.

### Comparison with results from wastewater monitoring

An alternative way to estimate excretion of specific compounds is wastewater monitoring, enabling estimation of LNCS usage. Fortunately, such sewage investigations have been performed in the same region by the TZW Karlsruhe shortly before the KarMeN study [61]. This allows direct comparison of results obtained either by biomonitoring or by wastewater monitoring. Indeed, the LNCS pattern found in the influent of two sewage treatment plants in the Karlsruhe area was very similar to our results. Main component was cyclamate with about 1000 t/year (extrapolated total input), followed by acesulfame and saccharin with 200–300 t/year. Only < 10 t/year sucralose was found, reflecting its low market share in 2004, shortly after the EU approval. Whereas saccharin and cyclamate are effectively removed upon sewage treatment, acesulfame and sucralose are also found in the effluent und thus in surface water, why acesulfame can even be used to monitor aquifer flows.

Similar wastewater investigations in other countries were recently summarized in [62]. This comparison reveals different LNCS consumption depending on the region. Results from Europe were similar to our results, showing high amounts of the three LNCS acesulfame, cyclamate and saccharin. However, sucralose amounts were remarkably different: as in our study, they were also low in Zürich, Switzerland (published 2009 [63]), but quite high in Athens, Greece (published 2013 [64]). Besides regional differences, growing market share seems to contribute, since a steep increase was observed between 2011 and 2012 in the Greek study. Outside Europe, considerably different LNCS patterns were reported for some regions [62, 65], whereas results from Australia resembled more the European situation [66].

### Contributing food groups

We observed plausible associations between urinary LNCS concentrations and the consumption of specific food groups, with table sweeteners and light soft drinks explaining the largest proportion of the observed variance. Consumption of table sweeteners was associated with urinary cyclamate and saccharin, while consumption of light soft drinks and Radler was associated with cyclamate and acesulfame. These LNCS combinations are common for these types of foods. Steviol glycosides were specifically associated with stevia sweetener and herbal & fruit teas, but they did not seem to be part of a LNCS mixture in any other food group. For fruit teas, stevia leaves are occasionally added for their “natural sweetness”. Although we were able to identify the major contributing food groups, a significant proportion of the observed variances for all quantified LNCS concentrations remained unexplained (53–71%). A potential reason are differences in the maximum permitted levels for LNCS in the EU, which are specific to each food group. These lower thresholds contribute to the overall measurable concentration of LNCS in urine, making it challenging to detect associations between LNCS and those food groups in our statistical model. Unexplained variance was highest for acesulfame, and lowest for cyclamate, but not substantially different among detected LNCS. This could be partly explained by uncertainties in the dietary assessment, such as recall bias and misreporting of food intake (both in terms of amount and type of food consumed). Furthermore, for food groups that include both LNCS-containing and LNCS-free foods, it is possible that this may result in a watered-down association with urinary concentrations, contributing to unexplained variances. We could not substantiate associations of aspartame traces with food consumption.

A comparably low intake for steviol glycoside may be related to sensoric issues, which limits the applicable amounts. To some extent, there are ongoing developments for taste improvements by using enzymatically modified products, e.g. in case of stevia [67].

### Estimation of LNCS intake

Literature-derived estimates of excretion rates were used for calculation of intake (compare methods section and Table S5). However, fundamental assumptions introduce a certain degree of uncertainty. For instance, the metabolization of absorbed cyclamate to cyclohexylamine, a metabolite with potential adverse health effects, is highly variable, ranging from 0 to 85% [68, 69]. Based on the work of Renwick [70], approximately 85% of individuals metabolize less than 1%, while about 4% metabolize more than 20% of a dose. We performed sensitivity analyses, calculating min/max scenarios of possible uncertainties for cyclamate (20–25% absorption/metabolization), steviol glycoside (20–48%), and saccharin (85–95%) (data not shown). The results were robust, supporting the observation that individuals who consume a high amount of foods containing LNCS have a significant exposure to combinations of LNCS.

For aspartame, advantame and neotame, we could not estimate a valid intake based on urinary concentrations. Quantification of dipeptide sweeteners such as aspartame is principally possible, but these are usually almost not detected in urine, since these LNCS are nearly completely hydrolysed in the gut [50]. Aspartame for instance is reported to be almost completely hydrolysed in the gut to yield aspartate, phenylalanine and methanol, which are then absorbed and enter normal endogenous metabolic pathways to be further metabolized [71]. The amounts of these digestion products are much lower than those obtained from other natural dietary sources [50]. We nevertheless observed traces of intact aspartame in 17% of the analysed urine samples, suggesting that a minor portion is absorbed and excreted unchanged in the urine. Assuming urinary aspartame recovery of 0.4%, our data suggest that the intake is in the same range as two of the most abundant LNCS, acesulfame and saccharin, which is plausible.

Both advantame and neotame are reported to be rapidly but incompletely absorbed. Their metabolites are primarily excreted through feces (> 80%), with urinary excretion representing a minor pathway [72, 73], either without prior absorption or following absorption and biliary excretion [74]. We were unable to quantify these metabolites as part of our urine analysis. Nonetheless, these LNCS also contribute to a mixed exposure and need to be taken into account when addressing the challenge of evaluating mixed LNCS exposure.

### Exposure to combinations of LNCS

Highest absolute excretion was observed for acesulfame, cyclamate, and saccharin followed by steviol glucuronide, which is in line with Buso et al. [41]. With regard to the exploitation of the respective ADI (compare Tables S5 and S8), cyclamate was the most relevant LNCS. Specific combinations are common. E.g., cyclamate is often used in combination with saccharin because the two substances enhance each other’s sweetening effects. Unfortunately, we were unable to quantify the specific exposure to aspartame. Based on the ingredient lists of processed foods on the German market [75] and data from the food control authority Karlsruhe [76], aspartame is a very common LNCS, for example, in soft drinks. Indeed, aspartame traces were frequently detected, including those participants with substantial mixed urinary excretion of other LNCS. Thus, we assume a relevant exposure for this LNCS as well, adding to the observed load of exposure to combinations of LNCS in our population and subsequently in other studies.

Whereas we were not able to quantify sucralose in 99% of the samples, it likely contributes to mixed LNCS exposure today since there has been a sharp increase in the use of sucralose in recent years [75]. Market share in Germany and application in the German food industry at the time of the study (2011-13) was probably still marginal after its authorization in the EU in 2004. Furthermore, sucralose is about 600 times sweeter than sucrose, leading to low concentrations in foods and subsequently not quantifiable concentrations in urine, considering also that sucralose is mainly excreted via feces. Absorption is reported to range from 8 to 22% in humans, followed by rapid and essentially unchanged excretion via urine [77]. The study of Buso et al. [41], which was conducted at the same time period, reported as well only marginal urinary sucralose concentrations.

The overall diet consists of a mix of various foods leading to a meaningful exposure to different LNCS, especially for a small proportion in our population. In a recent report from the German Federal Institute for Risk Assessment [78], the authors highlighted potential combination effects of certain LNCS, including sucralose, saccharin, and aspartame. In terms of toxicological relevance, the long-term daily intake of a mixture of LNCS may no longer be considered safe for health, given the assumed exposure amount of the respective ADI when used in combination. Many national and international policies and strategies have been implemented in recent years with the aim of reducing the amount of free sugars in processed foods. This is done specifically to reduce the risk of overweight, obesity, and associated non-communicable diseases. As a result, there has been an increasing use of LNCS [1]. This leads to a potentially increasing mixed exposure through the combination of various foods containing LNCS.

### Limitations and strengths

We utilized fundamental assumptions to estimate the intake of LNCS based on their urinary metabolites. This approach introduces a certain degree of uncertainty, especially for specific types of LNCS. However, it yields more accurate results for estimating LNCS exposure compared to exposure assessment based on self-reported food consumption such as 24-hour recalls which are prone to recall bias. Despite these limitations, we are confident to provide valid data on LNCS exposure and associated food sources. A clear strength is the estimation of LNCS intake based on 24 h urinary excretion of LNCS. Moreover, simultaneous quantification of several LNCS allows to infer a realistic exposure to the variety of commonly applied sweeteners, which is necessary for the urgent issue of risk assessment of combinations of LNCS in the daily diet.

## Conclusions

Combined LNCS use is very common for certain food groups such as table sweeteners or light soft drinks. In particular, individuals that are high consumers of certain processed foods are exposed to a notable mix of various LNCS via a combination of processed foods within the overall diet. Since data on mixed LNCS-health-associations are lacking, it is an urgent issue to evaluate the potential risk mixed LNCS intake rather than performing an isolated risk assessment.

## Electronic supplementary material

Below is the link to the electronic supplementary material.

Supplementary Material 1

## Data Availability

In accordance with the informed consent of the KarMeN-participants, pseudonymized study data could be provided to external researchers upon request for cooperation or data-use purposes. In this case, a cooperation or data use agreement must be concluded in advance. The use of KarMeN study data is therefore only possible with a detailed prior justification of its scientific purpose and after agreement of the Max Rubner-Institut.

## References

[1] Russell C, Baker P, Grimes C, Lindberg R, Lawrence MA (2022) Global trends in added sugars and non-nutritive sweetener use in the packaged food supply: drivers and implications for public health. Public Health Nutr 1–13. 10.1017/S1368980022001598 10.1017/S1368980022001598 PMC10346066 35899782

[2] Martyn D, Darch M, Roberts A, Lee HY, Yaqiong Tian T, Kaburagi N, Belmar P (2018) Low-/No-Calorie sweeteners: A review of global intakes. Nutrients 10(3). 10.3390/nu10030357 10.3390/nu10030357 PMC5872775 29543782

[3] Ashwell M, Gibson S, Bellisle F, Buttriss J, Drewnowski A, Fantino M, Gallagher AM, de Graaf K, Goscinny S, Hardman CA, Laviada-Molina H, Lopez-Garcia R, Magnuson B, Mellor D, Rogers PJ, Rowland I, Russell W, Sievenpiper JL, la Vecchia C (2020) Expert consensus on low-calorie sweeteners: facts, research gaps and suggested actions. Nutr Res Rev 33(1):145–154. 10.1017/S0954422419000283 31928558 10.1017/S0954422419000283 PMC7282854

[4] Rios-Leyvraz M, Montez J (2022) Health effects of the use of non-sugar sweeteners A systematic review and meta-analysis. World Health Organization, Geneva

[5] Use of non-sugar sweeteners (2023) WHO Guidline. World Health Organization Geneva 37256996

[6] Bundesinstitut für Risikobewertung (2014) Bewertung von Süßstoffen und Zuckeraustauschstoffen

[7] Cyclamate (Cyclamlc Acid, Calcium Cyclamate, and Food and Drug Administration, Cyclamate S (1980) Commissioner’s Decision

[8] Choudhary AK, Pretorius E (2017) Revisiting the safety of aspartame. Nutr Rev 75(9):718–730. 10.1093/nutrit/nux035 28938797 10.1093/nutrit/nux035

[9] Landrigan PJ, Straif K (2021) Aspartame and cancer - new evidence for causation. Environ Health 20(1):42. 10.1186/s12940-021-00725-y 33845854 10.1186/s12940-021-00725-y PMC8042911

[10] Riboli E, Beland FA, Lachenmeier DW, Marques MM, Phillips DH, Schernhammer E, Afghan A, Assunção R, Caderni G, Corton JC, De Aragão Umbuzeiro G, De Jong D, Deschasaux-Tanguy M, Hodge A, Ishihara J, Levy DD, Mandrioli D, McCullough ML, McNaughton SA, Morita T, Nugent AP, Ogawa K, Pandiri AR, Sergi CM, Touvier M, Zhang L, Benbrahim-Tallaa L, Chittiboyina S, Cuomo D, Debono NL, Debras C, De Conti A, El Ghissassi F, Fontvieille E, Harewood R, Kaldor J, Mattock H, Pasqual E, Rigutto G, Simba H, Suonio E, Viegas S, Wedekind R, Schubauer-Berigan MK, Madia F (2023) Carcinogenicity of aspartame, Methyleugenol, and isoeugenol. Lancet Oncol 24(8):848–850. 10.1016/s1470-2045(23)00341-8 37454664 10.1016/S1470-2045(23)00341-8

[11] Yin T, Li J, Wang Y, Liu K, Long T, Cheng L (2022) Artificially sweetened beverage consumption and cancer risk: A comprehensive Dose-Response Meta-Analysis of prospective studies. Nutrients 14(21). 10.3390/nu14214445 10.3390/nu14214445 PMC9658995 36364707

[12] Pavanello S, Moretto A, La Vecchia C, Alicandro G (2023) Non-sugar sweeteners and cancer: toxicological and epidemiological evidence. Regul Toxicol Pharmacol 139:105369. 10.1016/j.yrtph.2023.105369 36870410 10.1016/j.yrtph.2023.105369

[13] Debras C, Chazelas E, Sellem L, Porcher R, Druesne-Pecollo N, Esseddik Y, de Edelenyi FS, Agaesse C, De Sa A, Lutchia R, Fezeu LK, Julia C, Kesse-Guyot E, Alles B, Galan P, Hercberg S, Deschasaux-Tanguy M, Huybrechts I, Srour B, Touvier M (2022) Artificial sweeteners and risk of cardiovascular diseases: results from the prospective NutriNet-Sante cohort. BMJ 378:e071204. 10.1136/bmj-2022-071204 36638072 10.1136/bmj-2022-071204 PMC9449855

[14] Debras C, Deschasaux-Tanguy M, Chazelas E, Sellem L, Druesne-Pecollo N, Esseddik Y, Szabo de Edelenyi F, Agaesse C, De Sa A, Lutchia R, Julia C, Kesse-Guyot E, Alles B, Galan P, Hercberg S, Huybrechts I, Cosson E, Tatulashvili S, Srour B, Touvier M (2023) Artificial sweeteners and risk of type 2 diabetes in the prospective NutriNet-Sante cohort. Diabetes Care 46(9):1681–1690. 10.2337/dc23-0206 37490630 10.2337/dc23-0206 PMC10465821

[15] Diaz C, Rezende LFM, Sabag A, Lee DH, Ferrari G, Giovannucci EL, Rey-Lopez JP (2023) Artificially sweetened beverages and health outcomes: an umbrella review. Adv Nutr 14(4):710–717. 10.1016/j.advnut.2023.05.010 37187453 10.1016/j.advnut.2023.05.010 PMC10334147

[16] Espinosa A, Mendoza K, Laviada-Molina H, Rangel-Mendez JA, Molina-Segui F, Sun Q, Tobias DK, Willett WC, Mattei J (2023) Effects of non-nutritive sweeteners on the BMI of children and adolescents: a systematic review and meta-analysis of randomised controlled trials and prospective cohort studies. Lancet Glob Health 11(Suppl 1):S8. 10.1016/S2214-109X(23)00093-1 36866485 10.1016/S2214-109X(23)00093-1

[17] Tobiassen PA, Koster-Rasmussen R (2023) Substitution of sugar-sweetened beverages with non-caloric alternatives and weight change: A systematic review of randomized trials and meta-analysis. Obes Rev:e13652. 10.1111/obr.13652 10.1111/obr.13652 37880814

[18] Higgins KA, Mattes RD (2019) A randomized controlled trial contrasting the effects of 4 low-calorie sweeteners and sucrose on body weight in adults with overweight or obesity. Am J Clin Nutr 109(5):1288–1301. 10.1093/ajcn/nqy381 30997499 10.1093/ajcn/nqy381

[19] Steffen BT, Jacobs DR, Yi SY, Lees SJ, Shikany JM, Terry JG, Lewis CE, Carr JJ, Zhou X, Steffen LM (2023) Long-term aspartame and saccharin intakes are related to greater volumes of visceral, intermuscular, and subcutaneous adipose tissue: the CARDIA study. Int J Obes (Lond) 47(10):939–947. 10.1038/s41366-023-01336-y 37443272 10.1038/s41366-023-01336-y PMC10511315

[20] Buso MEC, Brouwer-Brolsma EM, Naomi ND, Ngo J, Soedamah-Muthu SS, Mavrogianni C, Harrold JA, Halford JCG, Raben A, Geleijnse JM, Manios Y, Serra-Majem L, Feskens EJM (2023) Sugar and low/no-calorie-sweetened beverage consumption and associations with body weight and waist circumference changes in five European cohort studies: the SWEET project. Eur J Nutr 62(7):2905–2918. 10.1007/s00394-023-03192-y 37407857 10.1007/s00394-023-03192-y PMC10468933

[21] Turner A, Veysey M, Keely S, Scarlett CJ, Lucock M, Beckett EL (2020) Intense sweeteners, taste receptors and the gut microbiome: A metabolic health perspective. Int J Environ Res Public Health 17(11). 10.3390/ijerph17114094 10.3390/ijerph17114094 PMC7312722 32521750

[22] Chometton S, Tsan L, Hayes AMR, Kanoski SE, Schier LA (2023) Early-Life influences of Low-Calorie sweetener consumption on sugar taste. Physiol Behav 114133. 10.1016/j.physbeh.2023.114133 10.1016/j.physbeh.2023.114133 PMC11062773 36801464

[23] Shum B, Georgia S (2021) The effects of Non-Nutritive sweetener consumption in the pediatric populations: what we know, what we don’t, and what we need to learn. Front Endocrinol (Lausanne) 12:625415. 10.3389/fendo.2021.625415 33868167 10.3389/fendo.2021.625415 PMC8049500

[24] Plows JF, Aris IM, Rifas-Shiman SL, Goran MI, Oken E (2022) Associations of maternal non-nutritive sweetener intake during pregnancy with offspring body mass index and body fat from birth to adolescence. Int J Obes (Lond) 46(1):186–193. 10.1038/s41366-021-00897-0 34611285 10.1038/s41366-021-00897-0 PMC8784986

[25] Li G, Wang R, Zhang C, Li L, Zhang J, Sun G (2022) Consumption of Non-Nutritive sweetener during pregnancy and weight gain in offspring: evidence from human studies. Nutrients 14(23). 10.3390/nu14235098 10.3390/nu14235098 PMC9739060 36501127

[26] Chiang YF, Chen HY, Lai YH, Ali M, Chen YC, Hsia SM (2022) Consumption of artificial sweetener acesulfame potassium increases preterm risk and uterine contraction with calcium influx increased via myosin light chain Kinase-Myosin light chain 20 related signaling pathway. Mol Nutr Food Res 66(20):e2200298. 10.1002/mnfr.202200298 35986687 10.1002/mnfr.202200298

[27] Lobach AR, Roberts A, Rowland IR (2019) Assessing the in vivo data on low/no-calorie sweeteners and the gut microbiota. Food Chem Toxicol 124:385–399. 10.1016/j.fct.2018.12.005 30557670 10.1016/j.fct.2018.12.005

[28] Schiffman SS, Nagle HT (2019) Revisited: assessing the in vivo data on low/no-calorie sweeteners and the gut microbiota. Food Chem Toxicol 132:110692. 10.1016/j.fct.2019.110692 31351100 10.1016/j.fct.2019.110692

[29] Roberts A, Lobach AR (2019) Response to the Letter to the Editor by S. Schiffman and H. Nagle: Revisiting the data and information that has collectively established the safety of low/no-calorie sweeteners, including sucralose. Food Chem Toxicol 132:110691. 10.1016/j.fct.2019.110691 10.1016/j.fct.2019.110691 31330167

[30] Conz A, Salmona M, Diomede L (2023) Effect of Non-Nutritive sweeteners on the gut microbiota. Nutrients 15(8). 10.3390/nu15081869 10.3390/nu15081869 PMC10144565 37111090

[31] Gauthier E, Milagro FI, Navas-Carretero S (2023) Effect of low-and non-calorie sweeteners on the gut microbiota: A review of clinical trials and cross-sectional studies. Nutrition 117:112237. 10.1016/j.nut.2023.112237 37897982 10.1016/j.nut.2023.112237

[32] Suez J, Cohen Y, Valdes-Mas R, Mor U, Dori-Bachash M, Federici S, Zmora N, Leshem A, Heinemann M, Linevsky R, Zur M, Ben-Zeev Brik R, Bukimer A, Eliyahu-Miller S, Metz A, Fischbein R, Sharov O, Malitsky S, Itkin M, Stettner N, Harmelin A, Shapiro H, Stein-Thoeringer CK, Segal E, Elinav E (2022) Personalized microbiome-driven effects of non-nutritive sweeteners on human glucose tolerance. Cell 185(18):3307–3328e3319. 10.1016/j.cell.2022.07.016 35987213 10.1016/j.cell.2022.07.016

[33] Green CH, Syn WK (2019) Non-nutritive sweeteners and their association with the metabolic syndrome and non-alcoholic fatty liver disease: a review of the literature. Eur J Nutr 58(5):1785–1800. 10.1007/s00394-019-01996-5 31119399 10.1007/s00394-019-01996-5

[34] Khan TA, Lee JJ, Ayoub-Charette S, Noronha JC, McGlynn N, Chiavaroli L, Sievenpiper JL (2023) WHO guideline on the use of non-sugar sweeteners: a need for reconsideration. Eur J Clin Nutr 77(11):1009–1013. 10.1038/s41430-023-01314-7 37723261 10.1038/s41430-023-01314-7 PMC10630128

[35] Hedrick VE, Nieto C, Grilo MF, Sylvetsky AC (2023) Non-sugar sweeteners: helpful or harmful? The challenge of developing intake recommendations with the available research. BMJ 383:e075293. 10.1136/bmj-2023-075293 37813435 10.1136/bmj-2023-075293 PMC11426959

[36] Regulation (EC) (2008) No 1333/2008 of the European Parliament and of the Council of 16 December 2008 on food additives. European Parliament and the Council of the European Union, Straßburg

[37] Logue C, Dowey LRC, Strain JJ, Verhagen H, McClean S, Gallagher AM (2017) Application of liquid Chromatography-Tandem mass spectrometry to determine urinary concentrations of five commonly used Low-Calorie sweeteners: A novel biomarker approach for assessing recent intakes?? J Agric Food Chem 65(22):4516–4525. 10.1021/acs.jafc.7b00404 28506059 10.1021/acs.jafc.7b00404

[38] Logue C, Dowey LRC, Verhagen H, Strain JJ, O’Mahony M, Kapsokefalou M, Athanasatou A, Gallagher AM (2020) A novel urinary biomarker approach reveals widespread exposure to multiple Low-Calorie sweeteners in adults. J Nutr 150(9):2435–2441. 10.1093/jn/nxaa184 32678445 10.1093/jn/nxaa184

[39] Zhang T, Gan Z, Gao C, Ma L, Li Y, Li X, Sun H (2016) Occurrence of artificial sweeteners in human liver and paired blood and urine samples from adults in Tianjin, China and their implications for human exposure. Environ Sci Process Impacts 18(9):1169–1176. 10.1039/c6em00130k 27383923 10.1039/c6em00130k

[40] Chu YY, Chen YH, Hsieh RH, Hsia SM, Wu HT, Chen YC (2022) Development and validation of the Chinese version Non-Nutritive sweetener food frequency questionnaire with urinary biomarker in children and adolescents. Public Health Nutr 25(8):1–23. 10.1017/S136898002200088X 10.1017/S136898002200088X PMC9991608 35414373

[41] Buso MEC, Boshuizen HC, Naomi ND, Maho W, Diepeveen-de Bruin M, Balvers MGJ, de Vries JHM, Harrold JA, Halford JCG, Raben A, Feskens EJM, Brouwer-Brolsma EM (2023) Relative validity of habitual sugar and low/no-calorie sweetener consumption assessed by FFQ, multiple 24-h dietary recalls and urinary biomarkers: an observational study within the SWEET project. Am J Clin Nutr. 10.1016/j.ajcnut.2023.11.019 38043866 10.1016/j.ajcnut.2023.11.019

[42] Bub A, Kriebel A, Dorr C, Bandt S, Rist M, Roth A, Hummel E, Kulling S, Hoffmann I, Watzl B (2016) The Karlsruhe metabolomics and nutrition (KarMeN) study: protocol and methods of a Cross-Sectional study to characterize the metabolome of healthy men and women. JMIR Res Protoc 5(3):e146. 10.2196/resprot.5792 27421387 10.2196/resprot.5792 PMC4967183

[43] Jakobsen J, Ovesen L, Fagt S, Pedersen AN (1997) para-aminobenzoic acid used as a marker for completeness of 24 hour urine: assessment of control limits for a specific HPLC method. Eur J Clin Nutr 51(8):514–519. 10.1038/sj.ejcn.1600434 11248876 10.1038/sj.ejcn.1600434

[44] Bingham S, Cummings JH (1983) The use of 4-aminobenzoic acid as a marker to validate the completeness of 24 h urine collections in man. Clin Sci (Lond) 64(6):629–635. 10.1042/cs0640629 6601560 10.1042/cs0640629

[45] Slimani N, Ferrari P, Ocke M, Welch A, Boeing H, Liere M, Pala V, Amiano P, Lagiou A, Mattisson I, Stripp C, Engeset D, Charrondiere R, Buzzard M, Staveren W, Riboli E (2000) Standardization of the 24-hour diet recall calibration method used in the European prospective investigation into cancer and nutrition (EPIC): general concepts and preliminary results. Eur J Clin Nutr 54(12):900–917 11114689 10.1038/sj.ejcn.1601107

[46] Slimani N, Deharveng G, Charrondiere RU, van Kappel AL, Ocke MC, Welch A, Lagiou A, van Liere M, Agudo A, Pala V, Brandstetter B, Andren C, Stripp C, van Staveren WA, Riboli E (1999) Structure of the standardized computerized 24-h diet recall interview used as reference method in the 22 centers participating in the EPIC project. European prospective investigation into cancer and nutrition. Comput Methods Programs Biomed 58(3):251–266 10094230 10.1016/s0169-2607(98)00088-1

[47] Slimani N, Casagrande C, Nicolas G, Freisling H, Huybrechts I, Ocké MC, Niekerk EM, Van Rossum C, Bellemans M, De Maeyer M, Lafay L, Krems C, Amiano P, Trolle E, Geelen A, De Vries JH, De Boer EJ (2011) The standardized computerized 24-h dietary recall method EPIC-Soft adapted for pan-European dietary monitoring. Eur J Clin Nutr 65(S1):S5–S15. 10.1038/ejcn.2011.83 21731006 10.1038/ejcn.2011.83

[48] Krüger R, Merz B, Rist MJ, Ferrario PG, Bub A, Kulling SE, Watzl B (2017) Associations of current diet with plasma and urine TMAO in the KarMeN study: direct and indirect contributions. Mol Nutr Food Res 61(11):1700363–na. 10.1002/mnfr.201700363 10.1002/mnfr.201700363 28755411

[49] Merz B, Frommherz L, Rist M, Kulling S, Bub A, Watzl B (2018) Dietary pattern and plasma BCAA-Variations in healthy men and Women—Results from the KarMeN study. Nutrients 10(5):623. 10.3390/nu10050623 29762522 10.3390/nu10050623 PMC5985475

[50] Magnuson BA, Carakostas MC, Moore NH, Poulos SP, Renwick AG (2016) Biological fate of low-calorie sweeteners. Nutr Rev 74(11):670–689. 10.1093/nutrit/nuw032 27753624 10.1093/nutrit/nuw032

[51] Report of the Scientific Committee for Food on Saccharin (1977) Report of the Scientific Committee for Food, vol 4

[52] Renwick AG, Williams RT (1972) The fate of cyclamate in man and other species. Biochem J 129(4):869–879. 10.1042/bj1290869 4655822 10.1042/bj1290869 PMC1174232

[53] Geuns JM, Buyse J, Vankeirsbilck A, Temme EH, Compernolle F, Toppet S (2006) Identification of steviol glucuronide in human urine. J Agric Food Chem 54(7):2794–2798. 10.1021/jf052693e 16569078 10.1021/jf052693e

[54] Younes M, Aquilina G, Castle L, Degen G, Engel KH, Fowler PJ, Frutos Fernandez MJ, Fürst P, Gundert-Remy U, Gürtler R, Husøy T, Manco M, Mennes W, Moldeus P, Passamonti S, Shah R, Waalkens‐Berendsen I, Wright M, Batke M, Boon P, Bruzell E, Chipman J, Crebelli R, Fitzgerald R, Fortes C, Halldorsson T, Leblanc JC, Lindtner O, Mortensen A, Ntzani E, Wallace H, Cascio C, Civitella C, Horvath Z, Lodi F, Mech A, Tard A, Vianello G (2022) Re‐evaluation of neohesperidine dihydrochalcone (E 959) as a food additive. EFSA J 20(11). 10.2903/j.efsa.2022.7595

[55] Ranney RE, Oppermann JA, Muldoon E, McMahon FG (1976) Comparative metabolism of aspartame in experimental animals and humans. J Toxicol Environ Health 2(2):441–451. 10.1080/15287397609529445 827618 10.1080/15287397609529445

[56] Box GE, Cox DR (1964) An analysis of transformations. J Roy Stat Soc: Ser B (Methodol) 26(2):211–243

[57] Azen R, Budescu DV (2003) The dominance analysis approach for comparing predictors in multiple regression. Psychol Methods 8(2):129–148 12924811 10.1037/1082-989x.8.2.129

[58] Judd CM, McClelland GH, Ryan CS (2011) Data analysis: A model comparison approach. Routledge

[59] Brown JD (2014) Multiple regression. Linear models in matrix form: A Hands-On approach for the behavioral sciences. Springer

[60] Diepeveen-de Bruin M, Maho W, Buso MEC, Naomi ND, Brouwer-Brolsma EM, Feskens EJM, Balvers MGJ (2023) Development and validation of a UPLC-MS/MS method for the quantification of sugars and non-nutritive sweeteners in human urine. J Chromatogr B Analyt Technol Biomed Life Sci 1225:123741. 10.1016/j.jchromb.2023.123741 37236072 10.1016/j.jchromb.2023.123741

[61] Scheurer M, Brauch HJ, Lange FT (2009) Analysis and occurrence of seven artificial sweeteners in German waste water and surface water and in soil aquifer treatment (SAT). Anal Bioanal Chem 394(6):1585–1594. 10.1007/s00216-009-2881-y 19533103 10.1007/s00216-009-2881-y

[62] Yue Y, Li L, Qu B, Liu Y, Wang X, Wang H, Chen S (2023) Levels, consumption, and variations of eight artificial sweeteners in the wastewater treatment plants of Dalian City, China. Sci Total Environ 892:163867. 10.1016/j.scitotenv.2023.163867 37201820 10.1016/j.scitotenv.2023.163867

[63] Buerge IJ, Buser HR, Kahle M, Muller MD, Poiger T (2009) Ubiquitous occurrence of the artificial sweetener acesulfame in the aquatic environment: an ideal chemical marker of domestic wastewater in groundwater. Environ Sci Technol 43(12):4381–4385. 10.1021/es900126x 19603650 10.1021/es900126x

[64] Kokotou MG, Thomaidis NS (2013) Determination of eight artificial sweeteners in wastewater by hydrophilic interaction liquid chromatography-tandem mass spectrometry. Anal Methods 5(16). 10.1039/c3ay40599k

[65] Subedi B, Kannan K (2014) Fate of artificial sweeteners in wastewater treatment plants in new York State, U.S.A. Environ Sci Technol 48(23):13668–13674. 10.1021/es504769c 25365516 10.1021/es504769c

[66] Li D, O’Brien JW, Tscharke BJ, Choi PM, Zheng Q, Ahmed F, Thompson J, Li J, Mueller JF, Sun H, Thomas KV (2020) National wastewater reconnaissance of artificial sweetener consumption and emission in Australia. Environ Int 143:105963. 10.1016/j.envint.2020.105963 32688159 10.1016/j.envint.2020.105963

[67] Samuel P, Ayoob KT, Magnuson BA, Wölwer-Rieck U, Jeppesen PB, Rogers PJ, Rowland I, Mathews R (2018) Stevia leaf to stevia sweetener: exploring its science, benefits, and future potential. J Nutr 148(7):1186S–1205S. 10.1093/jn/nxy102 29982648 10.1093/jn/nxy102

[68] Renwick AG, Thompson JP, O’Shaughnessy M, Walter EJ (2004) The metabolism of cyclamate to cyclohexylamine in humans during long-term administration. Toxicol Appl Pharmcol 196(3):367–380. 10.1016/j.taap.2004.01.013 10.1016/j.taap.2004.01.013 15094307

[69] Scientific Committee on Food (2000) Revised opinion on cyclamic acid and its sodium and calcium salts. European Commission, Brussels

[70] Renwick AG (1986) The metabolism of intense sweeteners. Xenobiotica 16(10–11):1057–1071. 10.3109/00498258609038983 3541395 10.3109/00498258609038983

[71] Scientific Opinion on the re- Evaluation of aspartame (E 951) as a food additive (2013). EFSA J 11 (12). 10.2903/j.efsa.2013.3496

[72] Ubukata K, Nakayama A, Mihara R (2011) Pharmacokinetics and metabolism of N-[N-[3-(3-hydroxy-4-methoxyphenyl) propyl]-alpha-aspartyl]-L-phenylalanine 1-methyl ester, monohydrate (advantame) in the rat, dog, and man. Food Chem Toxicol 49(Suppl 1):S8–29. 10.1016/j.fct.2011.06.042 22036030 10.1016/j.fct.2011.06.042

[73] Scientific Opinion of the Panel on Food Additives Flavourings, processing aids and materials in contact with food on a request from European commission on Neotame as a sweetener and flavour enhancer (2007). EFSA J 5 (11):581. 10.2903/j.efsa.2007.581

[74] Australia New Zealand Food Authority (2001) Attachment 3 - Safety Assessment Report Aplication A406 - Permission For Use Of Neotame. A406 - Inquiry Report and Regulatory Impact Statement

[75] Mintel Global New Product Database https://www.gnpd.com/

[76] Schorb S, Gleiss K, Wedekind R, Suonio E, Kull AK, Kuntz M, Walch SG, Lachenmeier DW (2023) Assessment of aspartame (E951) occurrence in selected foods and beverages on the German market 2000–2022. Foods 12(11). 10.3390/foods12112156 10.3390/foods12112156 PMC10252593 37297402

[77] Joint FAO, WHO Expert Committee on Food Additives (1989) Toxicological evaluation of certain food additives and contaminants / prepared by the 33rd meeting of the Joint FAO/WHO Expert Committee on Food Additives, Geneva, 21–30 March 1989. WHO food additives series, vol 24. Cambridge: Cambridge University Press

[78] German Federal Institute for Risk Assessment (BfR) (2023) [Do mixtures of multiple sweeteners pose health risks for humans?]. vol 005/2023. Berlin. 10.17590/20230207-071341

