# Supplementary material for: Urinary excretion of low- and no-calorie sweeteners (LNCS) and associated food sources, as observed in the German cross-sectional KarMeN-study
Source: Eur J Nutr. 2025 Mar 24;64(3):136. doi: 10.1007/s00394-025-03644-7 (PMC11933190; doi:10.1007/s00394-025-03644-7)
Supplement: Supplementary file 1 — Supplementary Material 1 [file 394_2025_3644_MOESM1_ESM.docx]

**Supplementary Material**

**Table S1:** Compound table

|  | Compound | RT | ISTD | Precursor | Fragments | | Cone | Collision | |
| --- | --- | --- | --- | --- | --- | --- | --- | --- | --- |
|  |  |  |  |  | 1 | 2 |  | 1 | 2 |
|  |  | [min] | # | [m/z] | [m/z] | [m/z] | [V] | [V] | [V] |
| 1 | Acesulfame | 1.20 | 11 | 162.4 | 82.1 | 78.0 | 35 | 15 | 25 |
| 2 | Steviol glucuronide | 3.09 | 18 | 493.5 | 317.5 | 113.2 | 40 | 25 | 25 |
| 3 | Cyclamate | 1.96 | 12 | 178.3 | 80.2 |  | 45 | 25 |  |
| 4 | Saccharin | 1.69 | 13 | 182.2 | 106.2 | 42.8 | 45 | 20 | 20 |
| 5 | Aspartame | 2.32 | 14 | 293.2 | 200.3 | 146.2 | 25 | 15 | 15 |
| 6 | Steviol | 3.55 | 18 | 317.5 | 317.5 |  | 60 | 15 |  |
| 7 | Neotame | 3.01 | 16 | 377.5 | 200.3 | 230.4 | 45 | 20 | 20 |
| 8 | Sucralose | 2.25 | 15 | 395.1 | 359.3 | 34.9 | 30 | 10 | 20 |
| 9 | Advantame | 2.76 | 16 | 457.4 | 200.2 | 244.2 | 45 | 20 | 15 |
| 10 | Neohesperidin DC | 2.62 | 17 | 611.5 | 303.3 | 327.1 | 65 | 35 | 30 |
| 11 | Acesulfame-d4 | 1.20 | n.d. | 166.4 | 86.1 |  | 35 | 15 |  |
| 12 | Cyclamate-d11 | 1.94 | n.d. | 189.3 | 80.2 |  | 45 | 25 |  |
| 13 | Saccharin-d4 | 1.68 | n.d. | 186.2 | 106.2 |  | 45 | 20 |  |
| 14 | Aspartame-d3 | 2.32 | n.d. | 296.2 | 200.3 |  | 25 | 15 |  |
| 15 | Sucralose-d6 | 2.25 | n.d. | 403.1 | 367.2 |  | 30 | 10 |  |
| 16 | Advantame-d3 | 2.76 | n.d. | 460.4 | 200.2 |  | 45 | 20 |  |
| 17 | Neohesperidin DC-d3 | 2.62 | n.d. | 614.5 | 306.3 |  | 65 | 35 |  |
| 18 | Glycocholic acid | 3.05 | n.d. | 464.2 | 464.3 |  | 70 | 13 |  |
| 19 * | Hesperetin DC | 2.87 | n.d. | 303.3 | 166.2 | 125.2 | 45 | 25 | 17 |

* not evaluated due to interferences

**Table S2:** Instrument, Software and Method Parameters

| **Parameter** | **Setting / value** | |
| --- | --- | --- |
| **UPLC parameters** | | |
| Instrument | Acquity H-Class UPLC | |
| Column | Acquity BEH C18, 130Å, 1.7 µm, 2.1 mm x 100 mm | |
| Pre-column | Acquity BEH C18 VanGuard, 130Å, 1.7 µm, 2.1 mm x 5 mm | |
| Eluent A | 5 mM aqueous ammonium acetate | |
| Eluent B | 7:3 mixture methanol and acetonitrile, 5 mM ammonium acetate | |
| Gradient table | | |
| *time [min]* | *Eluent A [%]* | *Eluent B [%]* |
| 0 | 94 | 6 |
| 2 | 5 | 95 |
| 3 | 5 | 95 |
| 3.1 | 94 | 6 |
| 6.0 | 94 | 6 |
| Flow rate | 0.4 mL/min | |
| Injection volume | 10 µL | |
| Column temperature | 40°C | |
| Autosampler temperature | 12°C | |
| **MS parameters** | | |
| Instrument | Xevo TQD triple quadrupole MS | |
| Ionization mode | ESI | |
| Polarity | negative | |
| Scan parameters | see compound table | |
| Desolvation temperature | 600°C | |
| Desolvation gas | 1000 L/h | |
| Cone gas | 40 L/h | |
| Source temperature | 110 °C | |
| Capillary voltage | 2.5 kV | |
| Solvent delay | 1 min | |
| **Software parameters** | | |
| Acquisition software | MassyLynx SCN855 | |
| Raw data processing | TargetLynx | |
| Peak integration | Apex track | |
| Smoothing | Mean; 2 times, smoothing width 2 | |
| Calibration | quadratic; weighting 1/x | |

**Supplemental Information S3:** Chemicals, sample preparation, calibration and validation

Chemicals

Acetonitrile and methanol (LC-MS grade) were obtained from VWR (Darmstadt, Germany). Ammonium formate (HPLC grade), formic acid (LC-MS grade) and most standards were from Sigma-Aldrich (Steinheim, Germany). Steviol and its glucuronide were from TRC (Toronto, Canada), supplied by Biozol (Eching, Germany), and hesperetin DC was from Extrasynthese (Lyon, France). Deuterated internal standards (ISTD) were purchased from Santa Cruz Biotechnology (Dallas, Texas, USA).

Sample preparation

Stock solutions and intermediate dilutions of standards and their isotope-labelled counterparts were prepared at 10 mM in 1:1 methanol/water or DMSO (steviol compounds). An eluent mixture 95%A / 5%B was used to achieve desired calibrator concentrations. Samples were prepared as follows: 10 µL ISTD mix (50µM), 10 µL urine sample and 20 µL eluent B were mixed, vortexed for 1 min and centrifuged at 14.800 rpm (21.000 x g) for 10 min at 5 °C. 20 µL were taken off and mixed with 80 µL eluent A, resulting in a combined dilution factor of 20. Vials were vortexed for 1 min, and 80 µL of the supernatant was transferred into a conic autosampler vial. The vial was centrifuged at 4750 rpm (4.500 x g) for 10 min at 8 °C to be ready for injection.

Calibration

Matrix-matched calibrators (7 levels) were prepared from 10 µL urine, 10 µL ISTD mix, 10 µL eluent B, and 10 µL STD mix to achieve desired concentrations (0 µM to 5 µM). Further sample preparation was identical to samples, leading to an upper quantification limit of 100 µM in the original urine sample (dilution factor 20). Consequently, samples with results >100 µM were diluted 1:10, and repeated accordingly (overall dilution factor 200). Controls (2 levels) were prepared by spiking either 20 µL or 60 µL STD mix (600 µM) to an end volume of 500 µL urine. 20 µL aliquots were frozen to be used as control samples. For each sequence, respective aliquots were thawed, 10 µL ISTD mix plus 10 µL eluent B were added, and prepared identical to study samples. Injected control samples (1 µM and 3 µM) correspond to 20 µM and 60 µM in the original urine samples, respectively.

In case low background signals were detected in urine matrix used for calibration, standard addition was performed to quantify the original analyte concentrations of this specific matrix sample. A second data processing run was performed, now using the real concentrations of the calibrators (matrix background + spike). These calibration curves were used for subsequent quantification of unknown samples.

**Table S4:** Description of initial food groups.

| **Food group** | **Foods included** |
| --- | --- |
| Wholegrain bread/rolls |  |
| White, brown or multigrain bread/rolls |  |
| Cereals and cereal products | Cereals, flours, rice and processed products like breakfast cereals, pasta, popcorn |
| Pastries | Cake, pies, savory pastries like filled puff pastries, cheese straw, snacks, peanut flips, cracker, tortilla chips |
| Vegetables and vegetable products |  |
| Mushrooms |  |
| Pulses | Including lentils, chickpeas, green peas, white/broad beans, kidney/soy beans; exception: green beans; incl. canned pulses and as sauce) |
| Potatoes and potato products | Fresh potatoes, heated and processed products like French fries, potato pancakes, potato chips |
| Fruit and fruit products (without juice) | Including unsweetened frozen fruit, fruit sauces and processed products like sweetened or heated fruit, canned fruit, dried fruit |
| Nuts and seeds | Hazelnuts, peanuts, almonds etc. or processed products like peanut butter, salted or roasted nuts |
| Animal fats | Including butter, lard, fish oil |
| Vegetable fats and oils | Including margarine and other spreadable fats (exception: in salad dressings) |
| Cheese and curd cheese | Including soft cheese, cream cheese, acid curd cheese |
| Milk and dairy products | Including cocoa drinks, milkshakes, yoghurt, (sour) cream, buttermilk, kefir, whey, milk powder |
| Eggs | Including scrambled, fried and boiled eggs, omelets, soufflé |
| Meat and meat products unsmoked | Including roast, goulash, schnitzel, beef olive, minced meat, meat balls, ground meat sauce |
| Sausages and meat products smoked | Including salami, liver sausage, ham, bacon, cured pork, meat loaf, bratwurst |
| Fish and fish products | Salt- and freshwater fish, shrimps, mussels, snails, processed products like caviar, tinned fish |
| Soups | Soups without stews |
| Sauces | Including warm and cold sauces (e.g. ketchup, salad dressing), mustard, vinegar etc.; exceptions: fruit-, vegetable-, ground meat sauce |
| Sweets | Including sweets with chocolate, confectionaries, candies, fruit gums, cereal bars |
| Ice cream |  |
| Sweet spreads | Jam, jellies, honey and chocolate spreads |
| Desserts | Creams and desserts including pudding, semolina, tiramisu |
| Water |  |
| Coffee and tea (black/green) |  |
| Herbal/fruit tea |  |
| Fruit juices/nectars | including spritzer |
| Soft drinks | e.g. lemonades, bitter lemon (incl. no/low-calorie drinks) |
| Other non-alcoholic beverages | e.g. malt coffee, malt beer, non-alcoholic beer, vegetable juices |
| Beer | including mixed beer drinks such as Radler |
| Wine and sparkling wine |  |
| Spirits | schnapps, liqueurs |
| Other alcoholic beverages | e.g. alcopops, cocktails |
| Miscellaneous | Milk substitutes (e.g. soy-based (milk, yoghurt, cheese), coconut-, rice-, oat-, almond drink, cereal drink with soy), meat substitutes (e.g. tofu, tempeh, soy protein, vegetarian sausages), cereal substitutes (e.g. soy flour/flakes, lupines), sweeteners, sugar substitutes, beverage powders/ -granules (e.g. cocoa powder, lemonade powder), herbs, spices, vegetarian spreads, protein powder, yeast, miso |

**Table S5:** Overview on analysed LNCS, their respective ADI, and estimated excretion rates

| **E-number** | **Name** | **ADI (mg/kg body weight / day)** | **Reference** | **Excretion rate (%)** | **Reference** |
| --- | --- | --- | --- | --- | --- |
| E 950 | Acesulfame K | 9 | [2] | 100 | [3] |
| E 951 | Aspartame | 40 | [4] | max. 0.4 - 4 | [5] |
| E 952 | Cyclamates | 7 | [6] | 22.5 | [7] |
| E 954 | Saccharins | 5 (sodium saccharin) 3.8 (expressed as acid) | [8] | 72 | [9,10] |
| E 955 | Sucralose | 15 | [11] | 15 | [12] |
| E 959 | Neohesperidine DC | 20 | [13] | 22 | [13] |
| E 960a | Steviol glycosides from Stevia | 4 | [14] | 34.5 | [15] |
| E 961 | Neotame | 2 | [16] | - |  |
| E 962 | Salt of aspartame-acesulfame | Respective ADIs  for E 950 & E 951 | [2,4] | Respective excretion rates for E950 & E951 |  |
| E 969 | Advantame | 5 | [17] | - |  |

**Table S6:** Validation data

| **Annotation** | **Acesulfame** | **Steviol Glucuronide** | **Cyclamate** | **Saccharin** | **Aspartame** | **Neotame** | **Sucralose** | **Advantame** | **Neohesperidin DC** |
| --- | --- | --- | --- | --- | --- | --- | --- | --- | --- |
| Sample Type | urine | urine | urine | urine | urine | urine | urine | urine | urine |
| Platform | LC-MS | LC-MS | LC-MS | LC-MS | LC-MS | LC-MS | LC-MS | LC-MS | LC-MS |
| Data Type | numeric | numeric | numeric | numeric | numeric | numeric | numeric | numeric | numeric |
| Analysis Mode | targeted | targeted | targeted | targeted | targeted | targeted | targeted | targeted | targeted |
| Unit | µmol/L | µmol/L | µmol/L | µmol/L | µmol/L | µmol/L | µmol/L | µmol/L | µmol/L |
| Calibration | internal | internal | internal | internal | internal | internal | internal | internal | internal |
| Weighting | 1/x | 1/x | 1/x | 1/x | 1/x | 1/x | 1/x | 1/x | 1/x |
| Standard addition | no | yes | no | no | no | no | no | no | no |
| LOD | 0.094 | 0.013 | 0.004 | 0.147 | 0.022 | 0.001 | 0.163 | 0.005 | 0.005 |
| LOQ | 0.312 | 0.044 | 0.013 | 0.491 | 0.075 | 0.003 | 0.543 | 0.016 | 0.015 |
| LLOQ | 1.75 | 1.85 | 1.99 | 2.73 | 1.63 | 2.33 | 7.47 | 1.98 | 1.81 |
| ULOQ | 195 | 193 | 196 | 194 | 192 | 162 | 173 | 194 | 194 |
| CV low (batch) | 3.1 | 3.8 | 4.0 | 5.5 | 7.4 | 5.1 | 10.3 | 4.0 | 4.2 |
| CV high (batch) | 5.0 | 5.5 | 5.2 | 5.4 | 5.8 | 6.0 | 8.3 | 4.1 | 6.3 |
| Accuracy (Bias %) low (batch) | 8.3 | 17.8 | 11.7 | 17.3 | 15.3 | 28.0 | 24.1 | 15.5 | 23.3 |
| Accuracy (Bias %) high (batch) | 10.9 | 21.9 | 15.0 | 15.2 | 11.2 | 18.2 | 17.4 | 16.4 | 25.1 |
| n_QC_Samples/Level (batch) | 12 | 12 | 12 | 12 | 12 | 12 | 12 | 12 | 12 |
| n_QC_Analyses/Level (batch) | 12 | 12 | 12 | 12 | 12 | 12 | 12 | 12 | 12 |
| CV low (day to day) | 8.4 | 11.7 | 10.0 | 9.5 | 9.7 | 9.4 | 16.5 | 8.5 | 9.9 |
| CV high (day to day) | 5.4 | 9.1 | 8.5 | 8.0 | 7.3 | 6.3 | 13.1 | 7.1 | 7.1 |
| Accuracy (Bias %) low (day to day) | 9.4 | 6.3 | 5.4 | -8.3 | -0.1 | 7.4 | -10.1 | -2.2 | 1.9 |
| Accuracy (Bias %) high (day to day) | 13.7 | 15.7 | 9.0 | -4.2 | 11.9 | 9.9 | 9.8 | 2.2 | 6.1 |
| n_QC_Samples/Level (day to day) | 10 | 10 | 10 | 10 | 10 | 10 | 10 | 10 | 10 |
| n_QC_Analyses/Level (day to day) | 20 | 20 | 20 | 20 | 20 | 20 | 20 | 20 | 20 |
| Recovery_low (%) | 91.4 | 79.3 | 92.2 | 96.2 | 90.3 | 82.7 | 79.3 | 82.4 | 76.3 |
| Recovery_high (%) | 82.7 | 79.0 | 84.7 | 92.8 | 83.8 | 82.2 | 82.6 | 82.2 | 76.1 |
| Matrix_Effect_low (%) | 110.9 | 118.9 | 96.5 | 52.5 | 104.1 | 120.7 | 189.9 | 139.4 | 110.5 |
| Matrix_Effect_high (%) | 109.6 | 115.6 | 96.5 | 57.4 | 98.9 | 120.6 | 117.5 | 121.0 | 101.5 |
| Recovery_Matrix_Effect_combined_low (%) | 101.4 | 94.2 | 89.0 | 50.5 | 94.0 | 99.9 | 150.5 | 114.9 | 84.3 |
| Recovery_Matrix_Effect_combined_high (%) | 90.6 | 91.3 | 81.7 | 53.2 | 82.8 | 99.2 | 97.1 | 99.5 | 77.2 |

**Table S7:** Urinary LNCS excretion (in mg/day) in total and stratified by quartiles of total urinary LNCS excretion

| **Variable** | **N=301** | **Q1 (n=75)** | **Q2 (n=75)** | **Q3 (n=76)** | **Q4 (n=75)** |
| --- | --- | --- | --- | --- | --- |
| Acesulfame | 0.00 (0.00-1.02) | 0.00 (0.00-0.00) | 0.00 (0.00-0.00) | 0.24 (0.00-1.36) | 4.04 (0.00-14.71) |
| Steviol glucuronide | 0.00 (0.00-0.00) | 0.00 (0.00-0.00) | 0.00 (0.00-0.00) | 0.00 (0.00-0.30) | 0.00 (0.00-0.00) |
| Cyclamate | 0.03 (0.01-0.26) | 0.01 (0.00-0.03) | 0.02 (0.01-0.05) | 0.03 (0.01-0.10) | 6.51 (0.18-16.32) |
| Saccharin | 0.00 (0.00-0.63) | 0.00 (0.00-0.00) | 0.00 (0.00-0.28) | 0.21 (0.00-0.74) | 2.64 (0.00-7.18) |
| Aspartame | 0.00 (0.00-0.00) | 0.00 (0.00-0.00) | 0.00 (0.00-0.00) | 0.00 (0.00-0.00) | 0.00 (0.00-0.00) |
| Neohesperidin DC | 0.00 (0.00-0.00) | 0.00 (0.00-0.00) | 0.00 (0.00-0.00) | 0.00 (0.00-0.00) | 0.00 (0.00-0.00) |

Values are given as median and interquartile range

**Table S8:** ADI exploitation of specific LNCS in the KarMeN study population

| **ADI exploitation** | **Acesulfame** | **Cyclamate** | **Steviol glycoside** | **Saccharin** | **Aspartame*** | **Neohesperidin DC** |
| --- | --- | --- | --- | --- | --- | --- |
| **<5%** | 286 | 260 | 293 | 293 | 301 | 301 |
| **>5%** | 8 | 16 | 3 | 5 | 0 | 0 |
| **>10%** | 5 | 12 | 4 | 1 | 0 | 0 |
| **>20%** | 2 | 7 | 1 | 2 | 0 | 0 |
| **>50%** | 0 | 3 | 0 | 0 | 0 | 0 |
| **>100%** | 0 | 3 | 0 | 0 | 0 | 0 |

*Exemplary scenario: 24h urinary excretion = 0.4% of aspartame intake

**References**

1. Logue C, Dowey LRC, Strain JJ, Verhagen H, McClean S, Gallagher AM (2017) Application of Liquid Chromatography-Tandem Mass Spectrometry To Determine Urinary Concentrations of Five Commonly Used Low-Calorie Sweeteners: A Novel Biomarker Approach for Assessing Recent Intakes? J Agric Food Chem 65 (22):4516-4525. doi:10.1021/acs.jafc.7b00404

2. Scientific Committee for Food (2000) Opinion - Re-evaluation of acesulfame K with reference to the previous SCF opinion of 1991. European Commission,, Brussels

3. Martyn D, Darch M, Roberts A, Lee HY, Yaqiong Tian T, Kaburagi N, Belmar P (2018) Low-/No-Calorie Sweeteners: A Review of Global Intakes. Nutrients 10 (3). doi:10.3390/nu10030357

4. Scientific Opinion on the re‐evaluation of aspartame (E 951) as a food additive (2013). EFSA Journal 11 (12). doi:10.2903/j.efsa.2013.3496

5. Ranney RE, Oppermann JA, Muldoon E, McMahon FG (1976) Comparative metabolism of aspartame in experimental animals and humans. J Toxicol Environ Health 2 (2):441-451. doi:10.1080/15287397609529445

6. Scientific Committee on Food (2000) Revised Opinion on Cyclamic Acid and its Sodium and Calcium Salts. European Commission, Brussels

7. Renwick AG, Williams RT (1972) The fate of cyclamate in man and other species. Biochemical Journal 129 (4):869-879. doi:10.1042/bj1290869

8. Scientific Committee for Food (1995) Opinion on Saccharin and its Sodium, Potassium and Calcium Salts. European Commission, Brussels, Belgium

9. Magnuson BA, Carakostas MC, Moore NH, Poulos SP, Renwick AG (2016) Biological fate of low-calorie sweeteners. Nutr Rev 74 (11):670-689. doi:10.1093/nutrit/nuw032

10. Report of the Scientific Committee for Food on Saccharin (1977). Report of the Scientific Committee for Food, vol 4.

11. Scientific Committee on Food (2000) Opinion of the Scientific Committee on Food on sucralose. European Commission, ,

12. Joint FAO/WHO Expert Committee on Food Additives (1989) Toxicological evaluation of certain food additives and contaminants / prepared by the 33rd meeting of the Joint FAO/WHO Expert Committee on Food Additives, Geneva, 21-30 March 1989. WHO food additives series, vol 24. Cambridge : Cambridge University Press,

13. Younes M, Aquilina G, Castle L, Degen G, Engel KH, Fowler PJ, Frutos Fernandez MJ, Fürst P, Gundert‐Remy U, Gürtler R, Husøy T, Manco M, Mennes W, Moldeus P, Passamonti S, Shah R, Waalkens‐Berendsen I, Wright M, Batke M, Boon P, Bruzell E, Chipman J, Crebelli R, Fitzgerald R, Fortes C, Halldorsson T, Leblanc JC, Lindtner O, Mortensen A, Ntzani E, Wallace H, Cascio C, Civitella C, Horvath Z, Lodi F, Mech A, Tard A, Vianello G (2022) Re‐evaluation of neohesperidine dihydrochalcone (E 959) as a food additive. EFSA Journal 20 (11). doi:10.2903/j.efsa.2022.7595

14. Scientific Opinion on the revised exposure assessment of steviol glycosides (E 960) for the proposed uses as a food additive (2014). EFSA Journal 12 (5). doi:10.2903/j.efsa.2014.3639

15. Geuns JM, Buyse J, Vankeirsbilck A, Temme EH, Compernolle F, Toppet S (2006) Identification of steviol glucuronide in human urine. J Agric Food Chem 54 (7):2794-2798. doi:10.1021/jf052693e

16. Scientific Opinion of the Panel on Food Additives, Flavourings, Processing Aids and Materials in Contact with Food on a request from European Commission on Neotame as a sweetener and flavour enhancer (2007). EFSA Journal 5 (11):581. doi:10.2903/j.efsa.2007.581

17. EFSA Panel on Food Additives and Nutrient Sources added to Food (2013) Scientific Opinion on the safety of advantame for the proposed uses as a food additive. EFSA Journal 11 (7):3301. doi:10.2903/j.efsa.2013.3301
